# Impact of Incomplete Coronary Revascularization on Late Ischemic and Bleeding Events after Transcatheter Aortic Valve Replacement

**DOI:** 10.3390/jcm9072267

**Published:** 2020-07-16

**Authors:** Adrien Carmona, Benjamin Marchandot, François Severac, Marion Kibler, Antonin Trimaille, Joe Heger, Marilou Peillex, Kensuke Matsushita, Jessica Ristorto, Viet Anh Hoang, Sébastien Hess, Laurence Jesel, Patrick Ohlmann, Olivier Morel

**Affiliations:** 1 Pôle d’Activité Médico-Chirurgicale Cardio-Vasculaire, Nouvel Hôpital Civil, Centre Hospitalier Universitaire, Université de Strasbourg, 67000 Strasbourg, France; adrien.carmona@chru-strasbourg.fr (A.C.); benjaminmarchandot@gmail.com (B.M.); marion.kibler@chru-strasbourg.fr (M.K.); antonin.trimaille@chru-strasbourg.fr (A.T.); joe.heger@chru-strasbourg.fr (J.H.); marilou.peillex@chru-strasbourg.fr (M.P.); matsuken_22@yahoo.co.jp (K.M.); ristorto-jess@hotmail.fr (J.R.); sebastien.hess@chru-strasbourg.fr (S.H.); Laurence.JESEL-MOREL@chru-strasbourg.fr (L.J.); patrick.ohlmann@chru-strasbourg.fr (P.O.); 2 Department of Biostatistics, Nouvel Hôpital Civil, Centre Hospitalier Universitaire, Université de Strasbourg, 67000 Strasbourg, France; Francois.severac@chru-strasbourg.fr; 3 Vietnam National Heart Institute, Bach Mai Hospital, Hanoi 100000, Vietnam; vietanhhoang78@yahoo.fr; 4 UMR INSERM 1260 Regenerative Nanomedicine, Université de Strasbourg, 67000 Strasbourg, France

**Keywords:** transcatheter aortic valve replacement, baseline SYNTAX score, residual SYNTAX score, bleeding

## Abstract

Background: The impact of coronary artery disease (CAD) and revascularization by percutaneous coronary intervention (PCI) on prognosis in patients undergoing transcatheter aortic valve replacement (TAVR) remain debated. A dismal prognosis in patients undergoing PCI has been associated with elevated baseline SYNTAX score (bSS) and residual SYNTAX score (rSS). The objective was to investigate whether the degree of bSS and rSS impacted ischemic and bleeding events after TAVR. Methods: bSS and rSS were calculated in 311 patients admitted for TAVR. The primary outcome was the occurrence of major adverse cardiac events (MACE), a composite endpoint of myocardial infarction, stroke, cardiovascular death, or rehospitalization for heart failure. The occurrence of late major/life-threatening bleeding complications (MLBCs) and each primary endpoint individually were the secondary endpoints. Results: bSS > 22 was associated with higher occurrence of MACE (*p* = 0.013). rSS > 8 and bSS > 22 had no impact on overall cardiovascular mortality. rSS > 8 and bSS > 22 were associated with higher rates of myocardial infarction (*p* = 0.001 and *p* = 0.004) and late occurrence of MLBCs. Multivariate analysis showed that bSS > 22 (sHR 2.48) and rSS > 8 (sHR 2.35) remained predictors of MLBCs but not of myocardial infarction. Conclusions: Incomplete coronary revascularization and CAD burden did not impact overall and cardiac mortality but constitute predictors of late MLBCs in TAVR patients.

## 1. Introduction

In elderly population, aortic stenosis (AS) coexists with significant coronary artery disease (CAD) in up to 50% of the cases and both diseases share common pathophysiological pattern associated with ageing including oxidative stress, endothelial dysfunction, enhanced inflammation, diabetes mellitus, or chronic kidney disease [1,2,3,4]. The noxious impact of CAD burden on survival in patients with AS was first suggested by surgical studies in which aortic valve replacement (AVR) combined with coronary artery bypass graft portended higher mortality risk than AVR alone in patients without CAD [5].

In patients scheduled for transcatheter aortic valve replacement (TAVR) procedures, current European Society of Cardiology (ESC) guidelines for myocardial revascularization suggest that percutaneous coronary intervention (PCI) should be considered before the index procedure in case of a coronary artery diameter stenosis of >70% affecting a proximal segment [6]. However, the evidence basis for such method remains limited and the impact of incomplete revascularization remains poorly investigated. Moreover, in this frail population characterized by a high bleeding risk, the noxious impact of subsequent antithrombotic therapies associated to PCI on bleedings events remains unexplored. Tailoring the antithrombotic therapy after TAVR is particularly challenging in this high-risk elderly population with significant overlap of both ischemic and bleeding events.

The residual SYNTAX score (rSS) is an angiographic score that assesses residual CAD burden after PCI [7]. A recent study [8] has established that staged PCI by achieving reasonable complete revascularization (rSS ≤ 8) improves mid-term survival and reduces the incidence of repeat PCI in patients with STEMI and multiple vessel disease. By contrast, residual CAD measured by higher rSS confers a worsened prognosis in patients undergoing PCI. In the present study, we sought to evaluate the impact of incomplete revascularization (rSS > 8) on late outcomes in TAVR patients including ischemic but also bleeding events.

## 2. Materials and Methods

397 patients were enrolled for TAVR with severe AS and high or intermediate surgical risk according to Logistic EuroScore at our institution (Nouvel Hôpital Civil, Université de Strasbourg, France) from November 2012 to December 2013 and then from June 2015 to June 2017. In all patients, aortic annulus diameter and area were determined using cardiac computerized tomography (CT). The aims of the study were explained to all participants and they gave their informed written consent before the procedure and agreed to anonymous processing of their data (France 2 Registry). The study was approved by the CNIL’s (Commission Nationale de l’Informatique et des Libertés) committee (ethical code number 911262). In the case of PCI, patients were pre-treated by P2Y12 inhibitors (mainly Clopidogrel), intravenous aspirin (125–250 mg), and 50–100 IU/kg of unfractionated heparin to target an ACT > 250 s. The use of GPIIbIIIa inhibitors, was left to the operators’ discretion.

Before the TAVR procedure, all patients received aspirin (75–160 mg) and Clopidogrel (loading dose 300 mg, 75 mg/day maintenance dose). The double antithrombotic therapy was ongoing after the procedure for 3 months. Only commercially available valves such as the Edwards SAPIEN XT or S3 prosthesis (Edwards Life sciences LLC, Irvine, CA, USA) and the CoreValve or Evolut-R (Medtronic CV, Irvine, CA, USA) were used as previously described. During the intervention, 100 international units/kg of unfractioned heparin were administered to achieve an activated clotting time of 250 to 350 s. At the end of the procedure, heparin was antagonized with protamine (100 UI/kg). All procedures were performed under analgesic sedation with Ultiva (remifentanil hydrochloride—0.10 to 0.15 microgram/kg/min).

### 2.1. Calculation of Baseline SYNTAX Score (bSS), Residual SYNTAX Score (rSS) and Syntax Revascularization Index (SRI)

The baseline SYNTAX Score (bSS) was calculated from the pre-procedural angiogram, in which each coronary lesion producing >50% diameter stenosis in vessels >1.5 mm by visual estimation was scored separately using the SS algorithm and added to obtain the overall SS. In patients with angiographic stenosis ≥70% or demonstrated residual ischemia assessed either by fractional flow reserve (FFR) or by perfusion myocardial tomography, a staged PCI was performed. The rSS was defined as the SS recalculated after staged PCI. The rSS was calculated in all patients enrolled in this study. The final post-PCI angiogram was scored to assess untreated disease after staged PCI and to calculate residual SYNTAX scores (rSS). Post-procedural angiograms were reviewed by a dedicated interventional cardiologist who was blinded to both baseline characteristics and clinical outcomes. Likewise, the Syntax revascularization index (SRI), an angiographic index tool designed to quantify the proportion of revascularized myocardium, was calculated and defined as: 100 (1—rSS/baseline SS) (%).

### 2.2. Collection of Data

Clinical outcomes were recorded and entered into a secure database. Follow-up information was obtained using a written questionnaire via a telephone interview with the cardiologist, referring physician or patient. In the absence of response, the patient’s electronic medical file was consulted. Endpoints were adjudicated by two physicians who were blinded to treatment allocation.

### 2.3. Study Endpoints

The primary endpoint was the major adverse cardiac event rate (MACE) defined as the composite of cardiovascular death, myocardial infarction (MI) (STEMI or NSTEMI or type 2 myocardial infarction), stroke, and rehospitalization for heart failure (HF). All clinical events were adjudicated by an events validation committee according to the VARC-2 criteria [9]. ST-segment elevation myocardial infarction (STEMI) was defined as a new ST-segment elevation in two consecutive leads with increased biochemical myocardial necrosis markers and non-ST-segment elevation myocardial infarction (NSTEMI) as the occurrence of ischemic symptoms associated with ST-segment depression or T-wave abnormalities and increased biochemical myocardial necrosis markers. Post-PCI troponin (Tn) elevations were not considered indicative of recurrent myocardial infarction. Stroke was defined as a focal loss of neurologic function caused by ischemic or hemorrhagic events with residual symptoms lasting >24 h. Secondary analyses were performed for each primary endpoint component.

The secondary endpoint was the occurrence of major bleeding and staged according to the BARC (Bleeding Academic Research Consortium) classification [10]. Major bleeding was defined as a BARC score ≥ Type 3b and minor bleeding as a BARC score < Type 3b.

### 2.4. Statistical Analysis

Categorical variables are all expressed as count and percentages. Continuous variables are reported as median and interquartile range (25th–75th). The normality of the distribution was assessed graphically with QQ (quantile-quantile) plots and using Shapiro–Wilk tests. Categorical variables were compared with a chi-square test or Fisher’s exact test. Continuous variables were compared using a non-parametric Mann–Whitney test. Event-free survival was calculated with the cumulative incidence function estimated using the competing risk approach of Kalbfleisch and Prentice [11]. Cumulative incidence was compared between rSS groups using the tests proposed by Gray [12]. Time to event was defined as the time from TAVR to the date of event, with patients censored at the end of the study and considering death as a competing risk. Multivariable survival analysis was realized using Fine and Gray’s sub-distribution hazard models. Variables with a *p*-value < 0.1 in univariate analyses were included in the multivariable model. To prevent expected collinearity between several variables (for instance CTADP post-TAVR >180 s and significant PVL at 1 month (Cramer’s V coefficient ¼ 0.51)), two separate multivariable analyses were performed. Results are presented as sub-distribution hazards ratios (sHR) with their 95% confidence intervals. All tests were 2-sided. A *p* value < 0.05 was considered significant. All the analyses were performed using R software version 3.6.0. R Core Team (2019). R: A language and environment for statistical computing. R Foundation for Statistical Computing, Vienna, Austria. URL https://www.R-project.org/.

## 3. Results

### 3.1. Patient Characteristics

**Figure 1:**
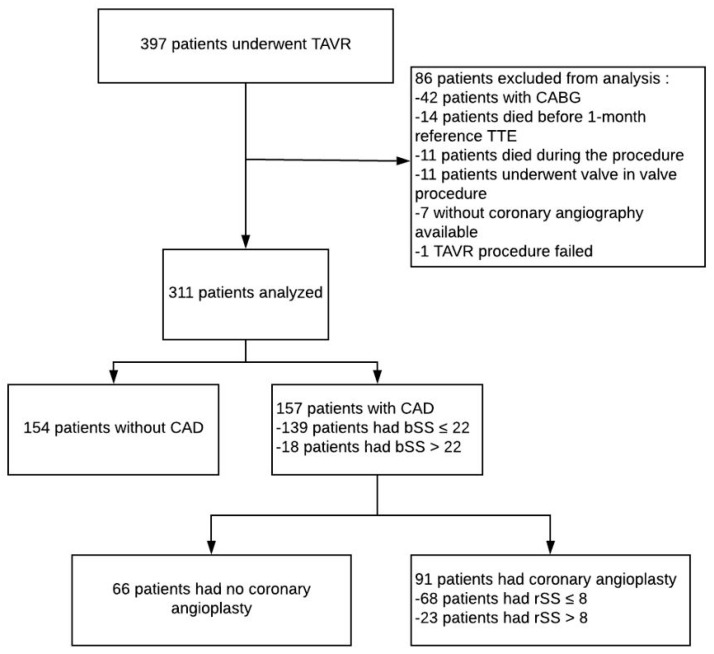
Flow chart of the study. bSS: baseline SYNTAX score, CABG: coronary artery bypass, CAD: coronary artery disease, CT-ADP: closure time adenosine diphosphate, rSS: residual SYNTAX score, TAVR: Transcatheter aortic valve replacement, TTE: transthoracic echocardiography.

From November 2012 to December 2013 and then from June 2015 to June 2017, 397 patients were admitted to our department (Nouvel Hôpital Civil, Strasbourg, France) for TAVR implantation. 86 patients in total were excluded from further analysis: 42 patients due to coronary artery bypass graft prior to TAVR, 14 patients died before 1-month follow up, 11 patients died during the procedure, 11 patients underwent valve in valve procedures, 7 patients had no coronary angiography data available and 1 TAVR procedure failed (Figure 1: Flow Chart).

bSS and rSS were calculated in 311 remaining patients. PCI was performed in 91/311 (29.3%) patients. Baseline characteristics according to bSS cut off value (≤ or >22) are shown in Supplementary Materials Table S1. Baseline characteristics according to rSS cut off value (≤ or >8) are shown in Table 1 and Table 2 bSS was higher in patients with incomplete revascularization (19 (13–25) vs. 0 (0–5), *p* < 0.001) translating a higher extent and complex CAD. As expected, PCI was more frequently performed in this subset of patient (57.9% vs. 24.5%; *p* < 0.001). Likewise, patients with incomplete revascularization were more likely to be on antiplatelet therapy and to present lower PRI value, a marker of the extent of P2Y_12_ inhibition (71 (60–78) vs. 63 (46–76); *p* = 0.023).

### 3.2. Clinical Outcomes

#### 3.2.1. Ischemic Events

Clinical outcomes were available for all patients with a median follow-up of 830 IQR (608–1032) days. Higher incidence of the composite endpoint could be evidenced in bSS > 22 patients (66.7% vs. 37.3%; *p* = 0.013). By contrast, the composite endpoint was not significantly higher in rSS > 8 patients with respect to rSS ≤ 8 patients.

Overall death, cardiac death, stroke, hospitalization for HF, and stroke did not differ significantly between groups, while higher rates of MI were observed in patients with incomplete revascularization (Table 3).

**Figure 2:**
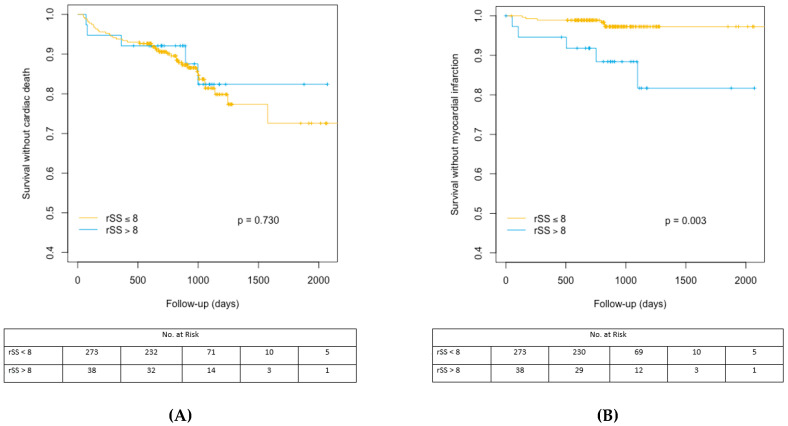
(**A**) Cumulative incidence analysis for the probability of cardiac survival according to rSS cut off value of 8. rSS: residual Syntax Score. (**B**) Cumulative incidence analysis for myocardial infarction-free survival after TAVR according to rSS cut off value of 8. rSS: residual SYNTAX Score, TAVR: Transcatheter aortic valve replacement.

The cumulative indicence curves representing the CV events–free and MI-free survival rates are represented in Figure 2A,B. Supplementary data regarding bSS and SRI cumulative incidence analyses for survival without CV death and MI are shown in Supplementary Materials Figure S1A,B and Supplementary Materials Figure S2A,B.

#### 3.2.2. Bleeding Events

**Figure 3:**
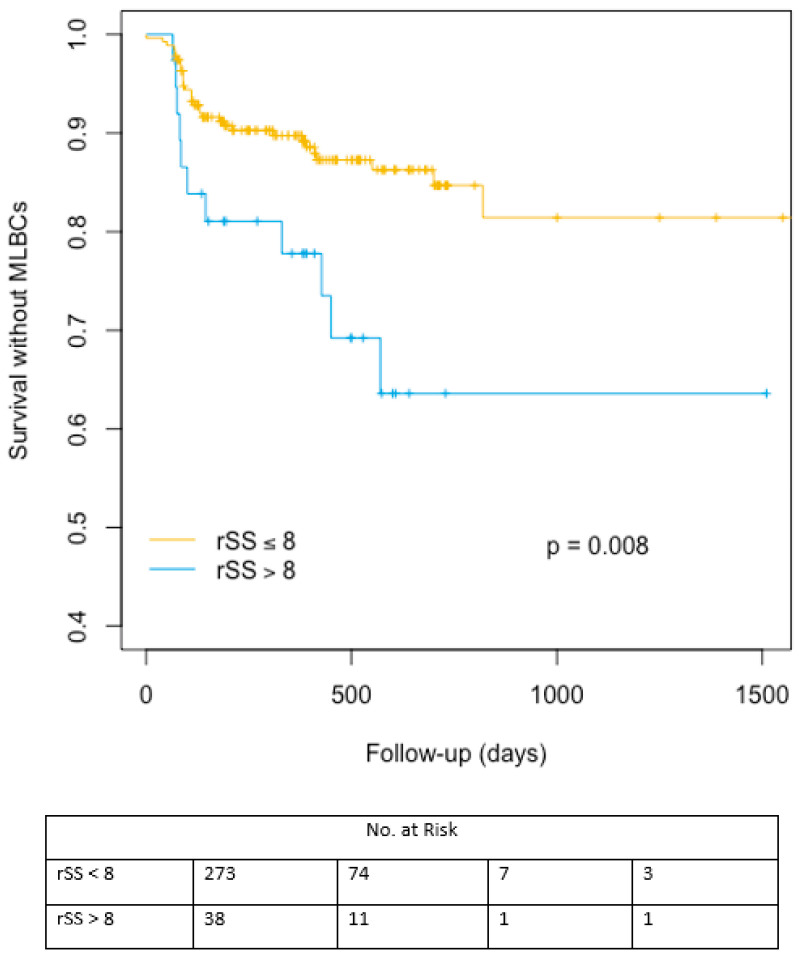
Cumulative incidence analysis for major/life-threatening bleeding events-free survival after TAVR according to rSS cut off value of 8. MLBCs: Major/life-threatening bleeding events-free survival, rSS: residual Syntax Score.

Significant increase in early but also late bleeding events was evidenced in patients with higher bSS or rSS. Likewise, late transfusion rates were higher in patients with rSS > 8. The cumulative indicence curves representing major/life-threatening bleeding events-free survival probability are represented in Figure 3. Supplementary data are available concerning bSS and SRI cumulative incidence analyses for major/life-threatening bleeding (MLBCs) events-free survival in Figure S3A,B.

#### 3.2.3. Myocardial Infarction Predictors

In univariate analysis, bSS > 22, rSS > 8, PCI, age, diabetes mellitus, cardiovascular disease heredity, chronic kidney disease, and PRI VASP (platelet reactivity index vasodilator stimulated phosphoprotein) post-TAVR were significant predictors for myocardial infarction occurrence. Age and PRI VASP post-TAVR were independent predictor of myocardial infarction (sHR: 0.917; 95% CI: 0.861 to 0.978; *p* = 0.008 and sHR: 0.970; 95% CI: 0.944 to 0.998; *p* = 0.033). No significant impact of cardiovascular risk factors, antiplatelet, and anticoagulant therapy duration or assignment on myocardial infarction could be established. Multivariate analysis identified age and PRI VASP post TAVR as the two independent predictors of MI occurrence after TAVR (Table 4).

#### 3.2.4. Predictors of Late Major/Life-Threatening Events

In univariate analysis, bleeding history, LVEF 1-month post TAVR, logistic EuroScore >20, paravalvular leak (PVL), bSS > 22, rSS > 8 and ongoing hemostasis disorders (CT ADP > 180 s) were significant predictors of MLBCs. No significant impact of Clopidogrel, aspirin, anticoagulant treatment allocation, or duration could be established on late bleeding events occurrence (Table 5).

Two multivariable models were performed owing to collinearity between CT-ADP > 180 s and significant PVL and were both strongly associated with MLBCs occurrence. In the two models, rSS > 8 and bSS > 22 remained a strong independent predictor of MLBCs (Table 6). Supplementary data regarding bSS analysis are shown in Table S2.

## 4. Discussion

The current report drawn from a cohort of 311 patients who underwent TAVR is the first study to specifically evaluate the impact of incomplete revascularization on thrombotic but also late bleeding events. The salient results of the present study are as follows: (1) incomplete revascularization (rSS > 8) was observed in a small proportion of the cohort and had no impact on overall and cardiac mortality. (2) Baseline CAD extent and incomplete revascularization were associated with increased MI rates. (3) Baseline CAD extent and incomplete revascularization were predictors of periprocedural bleedings and MLBCs regardless of anti-thrombotic treatment allocation or duration.

Altogether, our findings suggest that baseline CAD extent or incomplete revascularization, could identify a subset of patients with high bleeding diathesis after TAVR.

The first analysis of the eventual detrimental impact of CAD or non-revascularized myocardium on outcomes after TAVR showed no differences between groups on one-year all-cause mortality [13]. Although the analysis was made on a cohort of limited size (136 patients) and in the early stage of the TAVR era (2005–2007), the low rate of ischemic complication observed following TAVR has substantiated the view that the lowering of ischemic load afforded by PCI would not be mandatory in most of the cases. Likewise, in a cohort of similar size (124 TAVR patients), Van Mieghem and coworkers have emphasized that the completeness of the revascularization by PCI (32% of the cases) did not impact ischemic outcome [14]. More recent data by Stefanini and coworkers in a larger cohort (*n* = 445) have emphasized that both baseline and rSS were associated with enhanced rates of ischemic endpoint including cardiovascular death, stroke, and myocardial infarction, mostly driven by enhanced cardiac mortality. In this study, high rSS tertile (>14) was associated with the higher rate of ischemic endpoints at one year (no CAD 12.5%, low rSS: 16.5%, high rSS: 26.3; *p* = 0.043). Of interest, patients with lower rSS (0–14) had comparable outcome to that one observed with complete revascularization or no CAD suggesting that this threshold may constitute an acceptable extent of residual CAD after PCI [15]. This paradigm of an acceptable residual extent of CAD was recently challenged by the report by Shamekhi demonstrating a stepwise increase in 3-years mortality even observed for low rSS (no CAD 25.9%, low rSS (0–3) 31.4%, high rSS (>3) 41.5; *p* = 0.01) [16]. However, no association between CAD extend and MI rates, stroke, or major vascular complications could be observed at 30 days and longer follow-up endpoints were not investigated. In the present cohort, although a significant increase of MI could be evidenced in bSS > 22 or rSS > 8 sub-groups, no impact on overall or cardiac mortality could be established. The present findings are in line with the Italian CoreValve registry reporting similar one-year MACE (16.8%, 22.7%, 18.5%; *p* 0.594) and mortality (15.8%, 19.3%, 17.4%) rates in patients with complete, incomplete or no revascularization [17]. Altogether, these data suggest that the impact of initial or residual atherosclerotic coronary burden if existing appears to a very limited extent and did not impact patient survival.

In the setting of cardiac surgery, the detrimental role of concomitant CAD in patients with aortic stenosis (AS) has been described for many years. Although the combination of surgical aortic valve replacement (SAVR) and CABG increases the risk of periprocedural mortality as compared to the sole aortic valve replacement [18], there is also compelling evidence underlining that CABG in combination with SAVR reduces the long-term risk of myocardial infarction and mortality [19,20,21,22,23]. According to this view recent ESC guidelines recommend performing CABG with SAVR when a coronary artery lesion is ≥70% [6].

To the best of our knowledge, the impact of the SYNTAX score in patients treated with SAVR remains unexplored and little described. Regardless of the patient’s risk, large studies comparing TAVR and conventional SAVR have emphasized that the rate of recurrent myocardial infarction appeared comparable [24,25,26,27]. By contrast, important controversies remain concerning the extent of the bleeding risk. For instance, more bleeding was evidenced in PARTNER I in the SAVR group while SURTAVI showed no difference [24,27]. However, it should be emphasized that the direct comparison of the bleeding risk between SAVR and TAVR remains difficult since high heterogeneity in the antiplatelet treatment exists (DAPT vs. SAPT). Periprocedural bleeds may account for a substantial proportion of the risk in surgical procedures, whilst part of the bleeds in the TAVR group occurred at mid- and long-term follow-up as a possible consequence of ongoing DAPT or residual primary hemostasis disorders [28].

Another side effect of the liberal use of PCI in TAVR patients worth considering concerns the administration and the duration of dual antiplatelet therapy (DAPT). While DAPT is usually restricted to 3 months in TAVR patients, increased duration of DAPT up to 6 months is generally observed when PCI is performed. Safety concerns and the assessment of bleeding events are key elements when assessing any revascularization and antiplatelet strategy’s net beneficial effects. This crucial aspect was neglected in previous studies questioning the impact of revascularization strategies in TAVR. Several groups including ours have emphasized the high frequency and the noxious impact on mortality of the late bleeding after TAVR [29,30]. In that circumstance, improved patient care appears to be in the potential field of bleeding prevention. In patients treated by PCI, several studies have underlined the view that the Syntax Score could not be routinely used for the assessment of bleeding risk [31,32]. However, data from the large-scale ACUITY trial have established that high SS remained an independent predictor of 30-days major bleeding [33]. Accordingly, in a *post hoc* analysis of the PLATO trial, the extent of CAD was demonstrated to be an important determinant of the bleeding risk [34]. In line with this paradigm, the present study suggests that high bSS and rSS could behave as integrate markers of associated comorbidities or global patient sickness known to interfere with the bleeding risk in TAVR setting. The first hint demonstrating the paramount role of PVL in the determination of the bleeding risk was given by Genereux and co-workers. In the PARTNER cohort, the strongest predictor of bleeding events between 30 days and one year after TAVR was PVL [35]. Several recent studies demonstrated that HMW-multimers defects were induced by significant PVL that was associated with the increase of flow turbulences and the high shear stress forces [36,37]. This acquired primary hemostasis disorder was then proposed to explain why patients presenting PVL were more prone to bleed [38,39,40]. We have recently demonstrated that prolonged CT-ADP (>180 s), a surrogate marker of HMW-multimers Von Willebrand defect, as measured during the course of the procedure allows a very accurate identification of the presence of paravalvular leak in patients undergoing TAVR [41] but could also identify patients at higher risk of periprocedural but also late MLBCs [29]. In the present study, owing to the high collinearity between PVL, CT-ADP > 180s on one hand and bSS and RSS on the other hand, several models of multivariate analysis were built. In the different models, beyond the importance of PVL (or primary hemostasis disorders reflected by CT-ADP > 180s), initial and residual CAD extent could be pointed out as important determinants of the bleeding risk. The question of whether CAD burden acts primarily as a determinant of bleeding diathesis (i.e., calcifications) or represents only a marker of more intense or sustained anti-thrombotic strategies remains to be determined in dedicated studies. In our hand, we could not exclude that the lower PRI, as a marker of P2Y12 inhibition extent, value observed in patients with high bSS or rSS could have an impact on the rate of periprocedural complications. However, given the limited duration of DAPT in the present study, we do not believe that DAPT allocation could have substantially contributed to the enhanced risk of MLBCs.

### Study Limitations

This study displays several limitations. First, calculation of bSS and rSS are retrospective and assessed visually by an interventional cardiology expert without using computer software such as the quantitative coronary analysis system. This can cause a variation in the calculation of the Syntax score. Second, only a small proportion of patients with high rSS and bSS could be evidenced which may impact the interpretation of the data. Third, CT-ADP measurement was performed 24 h after TAVR only without being repeated during the follow-up. As a consequence, CT-ADP measurement could not be obtained at the time of the bleeding event. Fourth, anticoagulation treatment at the time of the bleeding event was unknown. Fifth, because of the little size of the cohort and the important proportion of the patients excluded from the analysis, multivariate analysis should be interpreted with caution, and the findings viewed as exploratory and hypothesis-generating. Regarding the number of myocardial infarction and MLBC events that occurred during the follow-up study period, we could not exclude a degree of overfitting in multivariate analyses as well as a loss of power to identify independent predictors. While these data limit to some extent the validity of our comparison, it must be emphasized that registries are mandatory for collecting real-life data on unselected patients.

## 5. Conclusions

Incomplete coronary revascularization and CAD burden did not impact overall and cardiac mortality but constituted predictors of late MLBCs in unselected TAVR patients.

## Figures and Tables

**Table 1:** Baseline characteristics according to residual SYNTAX score (rSS).

Variables	rSS ≤ 8	rSS > 8	*p* Value
**Clinical Parameters**			
Age (Median IQR)–year	85 (82–89)	85 (80–86)	0.127
Male sex–no./total no. (%)	124 (45.4%)	12 (31.6%)	0.107
EuroScore (Median IQR)–%	17 (11–25)	19(13–28)	0.263
BMI (Median IQR)	26.6 (23.4–30.0)	25.1 (22.0–26.9)	0.022
**NYHA Class before TAVR–no./total no. (%)**			
NYHA 2	81 (29.7%)	8 (21.1%)	0.271
NYHA 3	161 (59%)	24 (63.2%)	0.623
NYHA 4	31 (11.4%)	6 (15.8%)	0.429
**Cardiovascular Risk Factor and Medical History**			
Hypertension–no./total no. (%)	230 (84.2%)	31 (81.6%)	0.675
Diabetes mellitus–no./total no. (%)	97 (35.7%)	13 (34.2%)	0.861
Dyslipidemia–no./total no. (%)	151 (55.5%)	23 (60.5%)	0.56
Current smoking–no./total no. (%)	10 (3.7%)	2 (5.3%)	0.631
Current dialysis–no./total no. (%)	5 (1.8%)	2 (5.6%)	0.16
Family history of cardiovascular disease–no./total no. (%)	12 (4.4%)	3 (7.9%)	0.349
Previous angioplasty–no./total no. (%)	35 (12.8%)	11 (28.9%)	0.009
History of myocardial infarction–no./total no. (%)	37 (13.6%)	9 (23.7%)	0.102
History of atrial fibrillation–no./total no. (%)	116 (42.5%)	17 (44.72%)	0.793
Chronic kidney disease (serum creatinine > 150 µmol L)	59 (21.6%)	10 (26.3%)	0.513
Prior bleeding events–no./total no. (%)	34 (12.5%)	7 (18.4%)	0.308
**Pre-hospital Antithrombotic Management**			
Single APT–no./total no. (%)	147 (53.8%)	29 (76.3%)	0.009
Dual APT–no./total no. (%)	51 (18.7%)	19 (50%)	<0.001
Loading dose Clopidogrel–no./total no. (%)	123 (45.1%)	11 (28.9%)	0.06
Anticoagulant therapy–no./total no. (%)	113 (41.4%)	14 (36.8%)	0.593
**Imaging Parameters**			
Mean aortic gradient–mmHg ± DS	49.3 ± 12.9	45.5 ± 11.1	0.085
Aortic valve calcium score (Median IQR)–AU	2781.5 (2051–3806)	2713.0 (1790.5–3270.0)	0.399
CT aortic surface (Median IQR)–mm^2^ ± DS	476 (414–537)	436 (391–539)	0.386
**Echocardiography Parameters**			
LVEF pre-TAVR (Median IQR)–(%)	58 (50–64)	53 (40–63)	0.099
LVEF 1 month post-TAVR (Median IQR)–(%)	60 (54–66)	55 (48–65)	0.102
LVEDD (Median IQR)–mm ± DS	49 (45–54)	50 (44–56	0.646
LVESD (Median IQR)–mm ± DS	33 (28–39)	38 (30–43)	0.039
Outflow chamber of the left ventricle (Median IQR)–mm ± DS	21 (20–23)	21(20–23)	0.703
AVA baseline (Median IQR)–cm² ± DS	0.720 (0.590–0.860)	0.720 (0.552–0.867)	0.706
Mean Aortic Gradient (Median IQR)–mmHg ± DS	48 (41–58)	45 (39–51)	0.102
Systolic PAP (Median IQR)–mmHg ± DS	38 (30–47)	37 (31–44)	0.769
**Baseline Biological Characteristics**			
CT ADP Baseline (Median IQR)	186 (130–300)	175 (126–300)	0.533
CT ADP Day 1 Post TAVR (Median IQR)	121 (97–177)	141 (100–182)	0.388
PRI VASP Day 1 Post TAVR (Median IQR)	71 (60–78)	63 (46–76)	0.023
**Coronary AngiographyCharacteristics**			
Baseline SYNTAX score (bSS) (Median IQR)	0 (0–5)	19 (13–25)	<0.001
Residual SYNTAX score (rSS) (Median IQR)	0 (0–2)	13 (10–16)	<0.001
Angioplasty–no./total no. (%)	67 (24.5%)	22 (57.9%)	<0.001
Left main angioplasty–no./total no. (%)	6 (2.2%)	3 (7.9%)	0.05
Left anterior descending angioplasty–no./total no. (%)	39 (14.3%)	11 (28.9%)	0.021
Diagonal angioplasty–no./total no. (%)	2 (0.7%)	3 (7.9%)	0.001
Intermediate angioplasty–no./total no. (%)	2 (0.7%)	0 (0%)	0.597
Circumflex angioplasty–no./total no. (%)	14 (5.1%)	6 (15.8%)	0.012
Marginal angioplasty–no./total no. (%)	7 (2.6%)	2 (5.3%)	0.352
Right coronary artery angioplasty–no./total no. (%)	23 (8.4%)	8 (21.1%)	0.015

Data are presented as mean ± or n (%), APT: antiplatelet therapy, AVA: aortic valve area, BMI: body mass index, CT: computerized tomography, CT ADP: closure time adenosine diphosphate, DS: deviation standard, IQR: interquartile range, LVEDD: left ventricular end diastolic diameter, LVEF: left ventricular ejection fraction; LVESD: left ventricular end systolic diameter, NYHA: New York Heart Association, PAP: pulmonary artery pressure, PRI VASP: platelet reactivity index vasodilator stimulated phosphoprotein, rSS: residual SYNTAX score.

**Table 2:** Procedural TAVR characteristics according to residual SYNTAX score (rSS).

Variables	rSS ≤ 8	rSS > 8	*p* Value
**Valve**			
Sapien–no./total no. (%)	170 (62.3%)	26 (68.4%)	0.462
CoreValve–no./total no. (%)	103 (37.7%)	12 (31.6%)	0.462
Transfemoral approach–no./total no. (%)	247 (90.5%)	35 (92.1%)	0.746
**Size of the Introducer**			
14F	166 (60.8%)	27 (71.1%)	0.223
16F	41 (15%)	5 (13.2%)	0.762
18F	66 (24.2%)	6 (15.8%)	0.251
Reimpaction for significant paravalvular aortic regurgitation–no./total no. (%)	30 (11%)	4 (10.5%)	0.932
**Size Valve**			
23 mm–no./total no. (%)	83 (30.4%)	14 (36.8%)	0.422
26 mm–no./total no. (%)	103 (37.7%)	14 (36.8%)	0.916
29 mm–no./total no. (%)	76 (27.8%)	9 (23.7%)	0.59
31 mm–no./total no. (%)	11 (4%)	1 (2.6%)	0.675
**Discharge Antithrombotic Medication**			
ASA–no./total no. (%)	268 (98.2%)	35 (92.1%)	0.027
DAPT–no./total no. (%)	152 (55.7%)	27 (71.1%)	0.072
Clopidogrel–no./total no. (%)	151 (55.3%)	27 (71.1%)	0.066
Anticoagulant therapy–no./total no. (%)	121 (44.3%)	17 (44.7%)	0.962
No DAPT nor anticoagulant therapy–no./total no. (%)	7 (87.5%)	1 (12.5%)	0.948
Duration of DAPT (Median IQR)–Days	60 (60–90)	60 (37–90)	0.765

Data are presented as mean ± or n (%), ASA: acetylsalicylic acid, rSS: residual Syntax score, F: French, DAPT: dual antiplatelet therapy.

**Table 3:** Primary and secondary endpoints according to residual SYNTAX score (rSS) and baseline SYNTAX score (bSS).

Variables	rSS ≤ 8	rSS > 8	*p* Value	bSS ≤ 22	bSS > 22	*p* Value
**Primary Endpoint–no./total no. (%)**						
MACE = cardiovascular death and/or myocardial infarction and/or rehospitalization for heart failure and/or stroke	103 (37.9%)	18 (47.4%)	0.261	109 (37.3%)	12 (66.7%)	0.013
**Secondary Endpoint–no./total no. (%)**						
Death from any cause	87 (31.9%)	10 (26.3%)	0.489	92 (31.4%)	5 (27.8%)	0.748
Cardiovascular death	40 (14.7%)	5 (13.2%)	0.806	42 (14.3%	3 (16.7%)	0.785
Rehospitalization for heart failure	72 (26.4%)	11 (28.9%)	0.737	75 (25.6%)	8 (44.4%)	0.079
Myocardial infarction	7 (2.6%)	5 (13.2%)	0.001	9 (3.1%)	3 (16.7%)	0.004
Stroke	25 (9.2%)	4 (10.5%)	0.786	25 (8.5%)	4 (22.2%)	0.053
**Bleeding–no./total no. (%)**						
Immediate post-procedural major and life threating bleeding	54 (19.8%)	13 (34.2%)	0.043	60 (20.5%)	7 (38.9%)	0.065
Immediate post-procedural major bleeding	39 (14.3%)	13 (34.2%)	0.002	45 (15.4%)	7 (38.9%)	0.009
Immediate post-procedural life threatening bleeding	15 (5.5%)	0 (0%)	0.139	15 (5.1%)	0 (0%)	0.325
Bleeding requiring red blood cell transfusion >2 U	55 (20.1%)	12 (31.6%)	0.108	60 (20.5%)	7 (38.9%)	0.065
Late major and life threating bleeding	33 (12.1%)	11 (28.9%)	0.005	38 (13%)	6 (33.3%)	0.016
Late major bleeding	27 (9.9%)	8 (21.1%)	0.041	31 (10.6%)	4 (22.2%)	0.129
Late life-threatening bleeding	6 (2.2%)	1 (2.6%)	0.866	6 (2%)	1 (5.6%)	0.33
Late transfusion ≥ 2 U	32 (11.7%)	11 (28.9%)	0.004	38 (13%)	5 (27.8%)	0.077

Data are presented as mean ± or n (%), bSS: baseline Syntax Score, immediate: before than 30 days after TAVR, life threating bleeding: Bleeding in a critical organ, Late: more than 30 days after TAVR, MACE: Major adverse cardiac events, Major bleeding: Overt bleeding either associated with a drop in the hemoglobin level of at least 3.0 g/dL or requiring transfusion of two or three units, rSS: residual Syntax Score, TAVR: Transcatheter Aortic Valve Replacement, U: Unit.

**Table 4:** Predictors of myocardial infarction.

	Univariate			Multivariate		
Variables	sHR	CI 95%	*p* Value	sHR	CI 95%	*p* Value
**Coronary parameters**						
bSS > 22	6.318	1.724–23.150	0.005	1.416	0.147–13.677	0.764
rSS > 8	6.134	1.896–19.850	0.002	2.654	0.297–23.744	0.383
Angioplasty	6.283	1.681–23.482	0.006	1.654	0.319–8.571	0.589
Aortic valve calcium score	0.999	0.999–1.000	0.011			
**Clinical Parameters**						
Age	0.949	0.913–0.986	0.007	0.917	0.861–0.978	0.008
EuroScore > 20	2.509	0.714–8.815	0.151			
Male sex	0.713	0.210–2.420	0.588			
Body mass index (BMI)	1.056	0.980–1.139	0.154			
Hypertension	1.911	0.246–14.870	0.536			
Diabetes mellitus	3.281	0.965–11.160	0.057	5.502	0.936–32.325	0.059
Dyslipidemia	3.578	0.778–16.441	0.101			
Current smoking	2.941	0.453–19.062	0.258			
Cardiovascular disease heredity	4.453	0.900–22.029	0.067	11.940	0.842–169.272	0.067
Peripheral artery disease	1.124	0.298–4.246	0.863			
History of atrial fibrillation	1.700	0.522–5.538	0.379			
Chronic kidney disease (creatinine level >150 µmol.L)	2.856	0.881–9.265	0.080	2.968	0.508–17.347	0.227
COPD	0.945	0.207–4.320	0.942			
Stroke history	1.952	0.518–7.345	0.323			
Bleeding history	1.348	0.309–5.881	0.691			
**Echocardiography Parameters**						
LVEF pre TAVR	0.987	0.940; 1.037	0.611			
LVEF 1 month post TAVR	0.977	0.930; 1.025	0.342			
**Biological Parameters**						
CT ADP > 180	1.213	0.360–4.093	0.755			
PRI VASP Post TAVR	0.968	0.941–0.997	0.028	0.970	0.944–0.998	0.033
**Discharge Antithrombotic Medication**						
DAPT	3.251	0.709–14.910	0.129			
Clopidogrel	1.945	0.526–7.195	0.319			
Anticoagulant therapy	1.072	0.330–3.482	0.908			

Data are presented as mean ± or n (%), bSS: baseline SYNTAX score, COPD: chronic obstructive pulmonary disease, CT-ADP: closure time adenosine diphosphate, DAPT: dual antiplatelet therapy, sHR: sub-distribution hazard ratio, CI: confidence interval, LVEF: left ventricular ejection fraction, PRI VASP: platelet reactivity index vasodilator stimulated phosphoprotein, rSS: residual SYNTAX score.

**Table 5:** Predictors of late major/life-threatening bleeding complications.

	Univariate		
Variables	sHR	CI 95%	*p* Value
**Coronary Parameters**			
bSS > 22	2.704	1.179–6.203	0.019
rSS > 8	2.502	1.278–4.900	0.007
Angioplasty	1.502	0.819–2.754	0.188
**Clinical Parameters**			
Age	1.001	0.961–1.042	0.978
Aortic valve calcium score	1.006	0.976–1.036	0.716
EuroScore > 20	2.077	1.143–3.776	0.016
Male sex	1.123	0.622–2.028	0.700
Body mass index (BMI)	0.965	0.916–1.016	0.178
Hypertension	0.869	0.415–1.820	0.709
Diabetes mellitus	1.400	0.773–2.537	0.267
Peripheral artery disease	0.733	0.354–1.517	0.402
Bleeding history	2.100	1.079–4.086	0.029
Valve sapien	0.665	0.367–1.204	0.178
**Discharge Antithrombotic Medication**			
ASA	0.556	0.149–2.075	0.383
DAPT	1.175	0.643–2.147	0.600
Clopidogrel	0.887	0.492–1.600	0.691
Anticoagulant therapy	1.348	0.745–2.438	0.324
Duration of DAPT	1.002	0.998–1.005	0.362
**Biological Parameters**			
PVL >1/4 at 1 month follow up	29.090	10.334–81.911	<0.001
Post TAVR PRI VASP	0.989	0.969–1.011	0.625
CT ADP > 180	1.748	0.958–3.188	0.069
**Echocardiography Parameters**			
LVEF pre TAVR	0.960	0.941; 0.980	<0.001
LVEF 1 month post TAVR	0.974	0.951; 0.999	0.041

Data are presented as mean ± or n (%), ASA: acetylsalicylic acid, bSS: baseline SYNTAX score. CT-ADP: closure time adenosine diphosphate, DAPT: dual antiplatelet therapy, sHR: sub-distribution hazard ratio, CI: confidence interval, LVEF: left ventricular ejection fraction, PRI VASP: platelet reactivity index vasodilator stimulated phosphoprotein, PVL: paravalvular leak, rSS; residual SYNTAX score, TAVR: Transcatheter aortic valve replacement.

**Table 6:** Multivariate analysis of association between rSS with baseline characteristics and late major/ life-threatening bleeding (MLBCs) occurrence.

**Model 1: All Candidates Predictors Except Significant Post-TAVR PVL at 1 Month and bSS > 22**			
**Variable**	**Hazard Ratio**	**CI 95%**	***p* Value**
Residual SYNTAX score: rSS > 8	2.345	1.171–4.700	0.016
EuroScore > 20	2.081	1.102–3.931	0.024
CT-ADP > 180	3.302	1.257–4.216	0.007
Prior bleeding events	1.835	0.959–3.513	0.067
LVEF 1-month post-TAVR	0.975	0.953–0.998	0.034
**Model 2: All Candidates Predictors Except Post-TAVR CT-ADP > 180 s and bSS > 22**			
**Variable**	**Hazard Ratio**	**IC 95%**	***p* Value**
Residual SYNTAX score: rSS > 8	2.391	1.150–4.973	0.020
EuroScore > 20	1.703	0.882–3.288	0.113
PVL >1/4 at 1 month follow up	29.238	8.116–105.329	<0.001
Prior bleeding events	1.873	0.920–3.816	0.084
LVEF 1-month post-TAVR	0.985	0.959–1.012	0.276

bSS: baseline Syntax Score, CI: confidence interval, CT-ADP: closure time adenosine diphosphate, LVEF: left ventricular ejection fraction, rSS, residual SYNTAX score, PVL: paravalvular leak, TAVR: transcatheter aortic valve replacement.

## Supplementary Materials

The following are available online at https://www.mdpi.com/2077-0383/9/7/2267/s1, Figure S1A: Kaplan–Meier analysis for the probability of cardiac survival according to bSS cut off value of 22, Figure S1B: Kaplan–Meier analysis for myocardial infarction-free survival after TAVR according to bSS cut off value of 22, Figure S2A: Kaplan–Meier analysis for the probability of cardiac survival according to SRI cut off value of 80, Figure S2B: Kaplan–Meier analyses for myocardial infarction-free survival probability, according to SRI cut off value of 80, Figure S3A: Cumulative incidence analysis for Major/life threatening bleeding events-free survival probability according to bSS cut off value of 22; Figure S3B: Cumulative incidence analysis for Major/life threatening bleeding events-free survival probability according to SRI cut off value of 80; Table S1: Baseline characteristic according to according to baseline SYNTAX score (bSS), Table S2: Multivariate analysis of association between bSS with baseline characteristics and late major/ life-threatening bleeding (MLBCs) occurrence.
