# Supplementary material for: Impact of Incomplete Coronary Revascularization on Late Ischemic and Bleeding Events after Transcatheter Aortic Valve Replacement
Source: J Clin Med. 2020 Jul 16;9(7):2267. doi: 10.3390/jcm9072267 (PMC7408638; doi:10.3390/jcm9072267)
Supplement: Supplementary file 1 [file jcm-09-02267-s001.pdf]

Supplementary Materials

Table S1: Baseline characteristic according to according to baseline SYNTAX score (bSS)

Variables	bSS ≤ 22	bSS > 22	p Value
**Clinical parameters**			
Age (Median IQR) – yr.	85 (82-89)	84 (81-86)	0.056
Male sex - no./total no. (%)	128 (43.7%)	8 (44.4%)	0.950
EuroScore (Median IQR) - %	17 (11-25)	19 (11-28)	0.820
BMI (Median IQR)	26 (23-30)	26 (24-29)	0.936
**NYHA class before TAVR - no./total no. (%)**			
NYHA 2	87 (29.7)	2 (11.1%)	0.090
NYHA 3	171 (58.4%)	14 (77.8%)	0.103
NYHA 4	35 (11.9%)	2 (11.1%)	0.915
**Cardiovascular risk factor and history**			
Hypertension - no./total no. (%)	246 (84%)	15 (83.3%)	0.944
Diabetes mellitus - no./total no. (%)	103 (35.2%)	7 (38.9%)	0.756
Dyslipidemia - no./total no. (%)	160 (54.8%)	14 (77.8%)	0.057
Current smoking - no./total no. (%)	11 (3.8%)	1 (5.6%)	0.7
Current dialysis - no./total no. (%)	6 (2.1%)	1 (5.6%)	0.335
Cardiovascular disease heredity - no./total no. (%)	14 (4.8%)	1 (5.6%)	0.884
Prior angioplasty - no./total no. (%)	37 (12.6%)	9 (50%)	<0.001
History of myocardial infarction - no./total no. (%)	37 (12.7%)	9 (50%)	<0.001
History of atrial fibrillation - no./total no. (%)	125 (42.7%)	8 (44.4%)	0.882
Chronic kidney disease (creatinine level >150µmol.L)	63 (21.5%)	6 (33.3%)	0.241
Prior bleeding events - no./total no. (%)	36 (12.3%)	5 (27.8%)	0.059
**Pre hospital antithrombotic management**			
Single APT - no./total no. (%)	159 (54.3%)	17 (94.4%)	0.001
Dual APT - no./total no. (%)	58 (19.8%)	12 (66.7%)	< 0.001
Loading dose Clopidogrel - no./total no. (%)	130 (44.4%)	4 (22.2%)	0.066
Anticoagulant therapy - no./total no. (%)	120 (41%)	7 (38.9%)	0.863
**Imaging parameters**			
Mean Aortic Gradient (Median IQR) - mmHg	48 (40-58)	49 (13-51)	0.569
Aortic valve calcium score (Median IQR) - AU	2752 (2033-3709)	3018 (2547-4541)	0.187
CT Aortic surface (Median IQR) - mm^2	470 (410-536)	538 (503-636)	0.012
**Baseline biological characteristics**			
CT ADP Baseline (Median IQR)	185 (129-300)	214 (137-300)	0.332
CT ADP Post TAVR (Median IQR) - Day 1	121 (97-177)	149 (116-222)	0.117
PRI VASP Post TAVR (Median IQR) - Day 1	71 (58-78)	67 (46-70)	0.065
***Coronary angiography* characteristics**			
Baseline SYNTAX Score (bSS) (Median IQR)	0 (0-7)	27 (25-29)	< 0.001
Residual SYNTAX Score(rSS) (Median IQR)	0 (0-3)	13 (9-19)	<0.001
Angioplasty — no./total no. (%)	72 (24.6%)	17 (94.4%)	<0.001
Left Main Angioplasty — no./total no. (%)	4 (1.4%)	5 (27.8%)	<0.001
Left anterior descending Angioplasty — no./total no. (%)	38 (13%)	12 (66.7%)	<0.001
Diagonal Angioplasty — no./total no. (%)	3 (1%)	2 (11.1%)	0.001
Intermediate Angioplasty — no./total no. (%)	2 (0.7%)	0 (0%)	0.725
Circumflex Angioplasty — no./total no. (%)	13 (4.4%)	7 (38.9%)	<0.001
Marginal Angioplasty — no./total no. (%)	7 (2.4%)	2 (11.1%)	0.032
Right coronary artery Angioplasty — no./total no. (%)	25 (8.5%)	6 (33.3%)	0.001

Data are presented as mean ± or n (%), APT : Antiplatelet therapy, BMI : Body mass index, CT : computerized tomography, CT ADP : closure time adenosine diphosphate, NYHA : New York Heart Association, PRI VASP : platelet reactivity index vasodilator stimulated phosphoprotein, rSS : residual Syntax Score.

Table S2: Multivariate analysis of association between bSS with baseline characteristics and late major/ life-threatening bleeding (MLBCs) occurrence

**Model 1 : all candidates predictors except significant post-TAVR PVL at 1 month and rSS > 8**			
**Variable**	**Hazard Ratio**	**CI 95%**	p Value
Baseline SYNTAX score : bSS > 22	2.477	1.018-6.023	0.046
EuroScore > 20	2.161	1.126-4.148	0.021
CT-ADP > 180	2.206	1.197-4.065	0.011
Prior bleeding events	1.742	0.906-3.351	0.096
LVEF 1 month post TAVR	0.976	0.953-0.999	0.038
**Model 2 : all candidates predictors except post-TAVR CT-ADP > 180 s and rSS > 8**			
Variable	Hazard Ratio	IC 95%	p Value
Baseline SYNTAX score : bSS > 22	2.584	1.001-6.668	0.049
EuroScore > 20	1.806	0.919-3.548	0.087
PVL >1/4 at 1 month follow up	26.539	7.329-96.102	< 0.001
Prior bleeding events	1.837	0.921-3.664	0.084
LVEF 1 month post TAVR	0.985	0.958-1.012	0.280

bSS : baseline Syntax Score, CI : confidence interval, CT ADP : closure time adenosine diphosphate, LVEF : Left ventricular ejection fraction, rSS, residual Syntax Score, PVL : paravalvular leak, TAVR : Transcatheter aortic valve replacement.

Figure S1A : Cumulative incidence analysis for the probability of cardiac survival according to bSS cut off value of 22

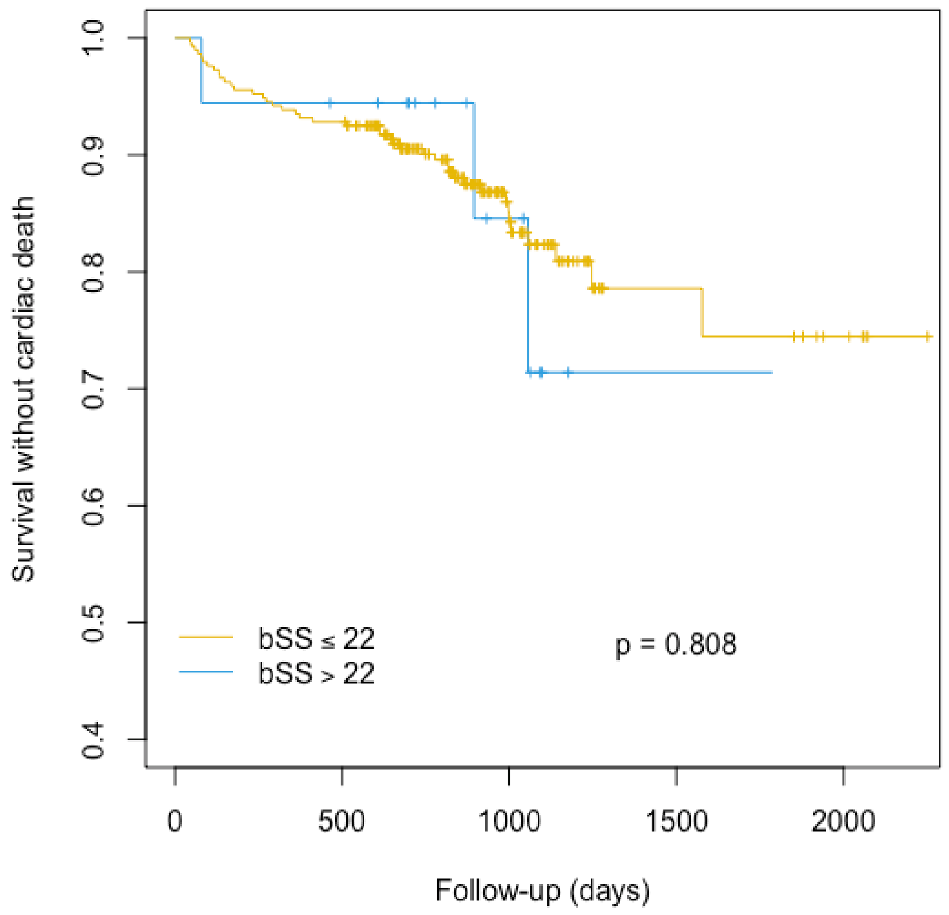

No. at Risk

bSS < 22	293	249	78	12	6
bSS > 22	18	15	7	1	0

Figure legends : bSS : baseline Syntax Score

Figure S1B : Cumulative incidence for myocardial infarction-free survival after TAVR according to bSS cut off value of 22

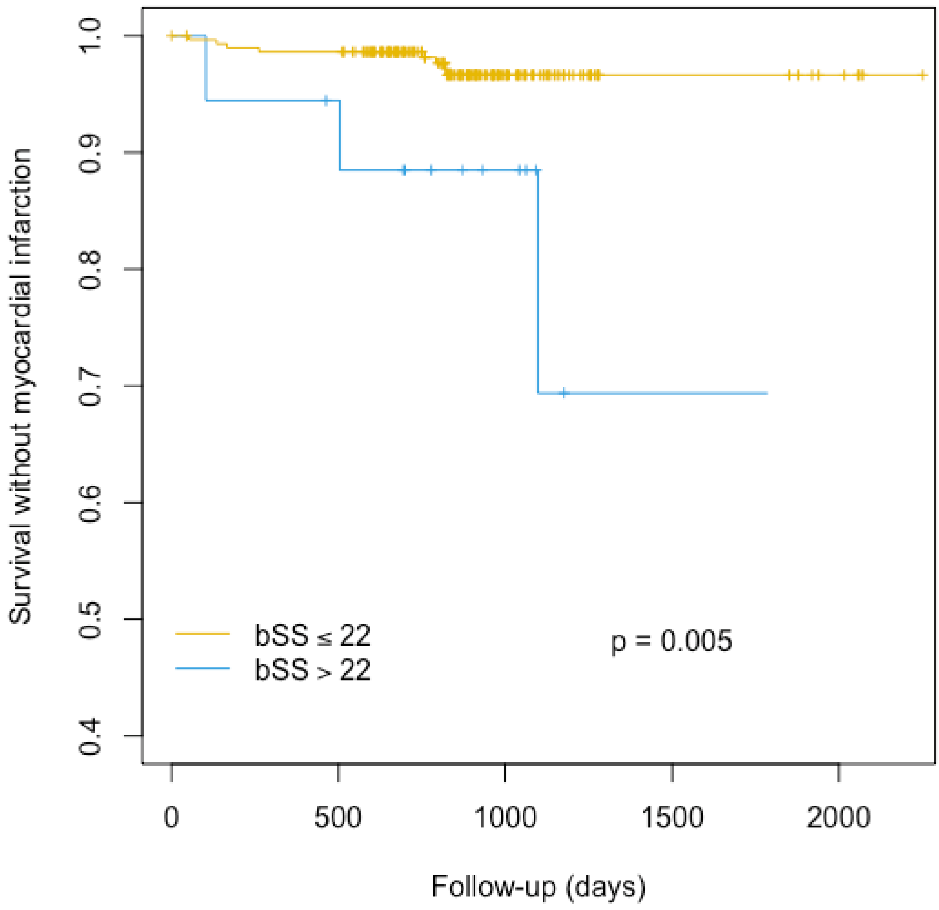

No at Risk					
bSS < 22	293	245	74	12	6
bSS > 22	18	14	7	1	0

Figure legends : bSS : baseline Syntax Score

Figure S2A : Cumulative incidence analysis for the probability of cardiac survival according to SRI cut off value of 80

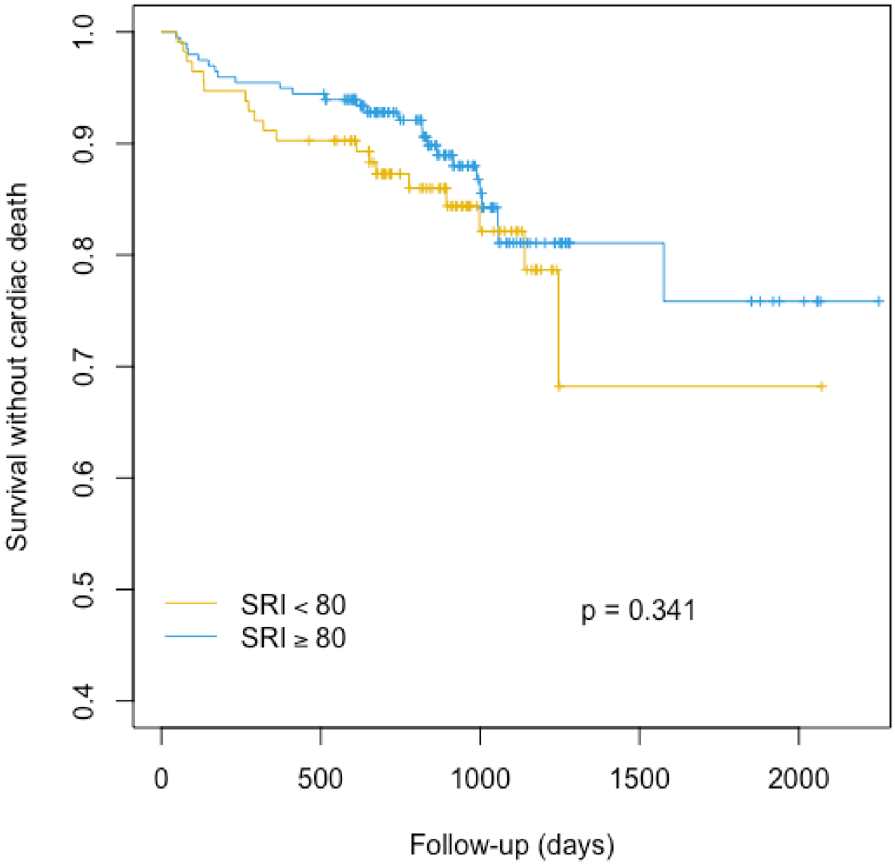

No. at Risk					
SRI < 80	113	91	30	2	1
SRI > 80	198	173	55	11	5

Figure legends : SRI : Syntax Revascularisation Index

Figure S2B : Cumulative incidence analyses for myocardial infarction-free survival probability according to SRI cut off value of 80

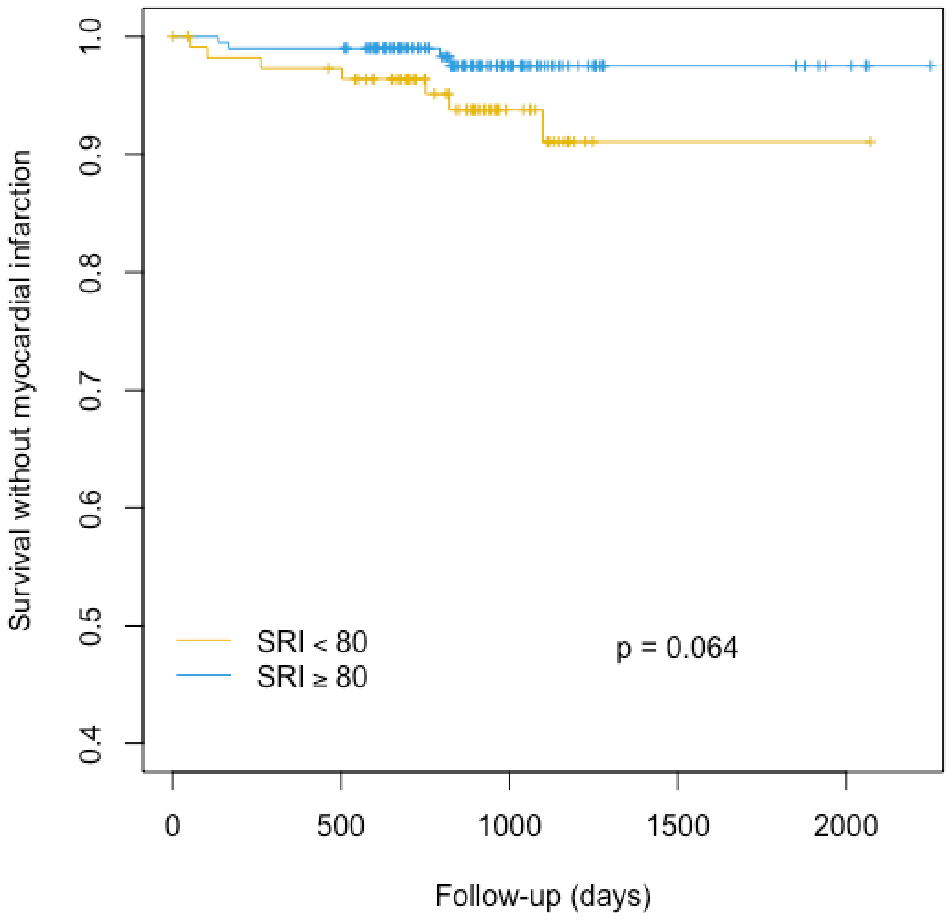

No. at Risk	0	500	1000	1500	2000
SRI < 80	113	87	27	2	1
SRI > 80	198	172	54	11	5

Figure legends : SRI : Syntax Revascularisation Index

Figure S3A : Cumulative incidence analysis for Major/life threatening bleeding events-free survival probability according to bSS cut off value of 22

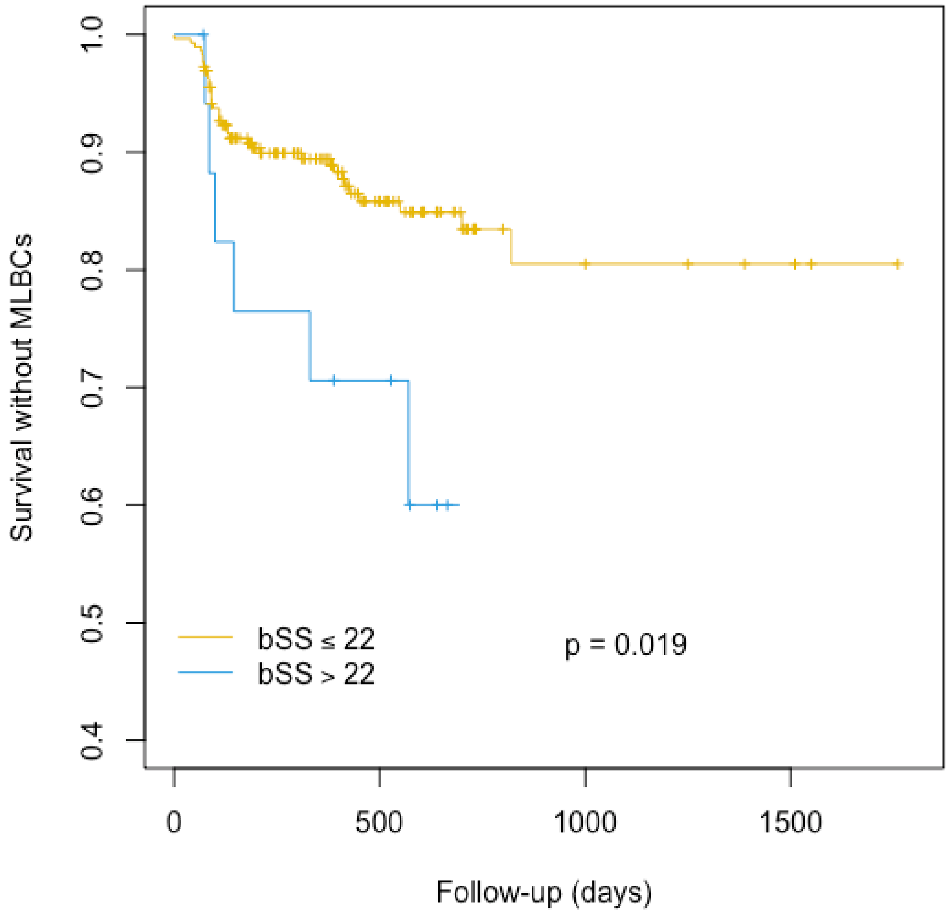

No at Risk				
bSS < 22	0	35	38	38
bSS > 22	0	5	6	6

Figure legends : bSS : baseline Syntax Score; MLBCs : Major/life threatening bleeding

Figure S3B : Cumulative incidence analysis for Major/life threatening bleeding events-free survival probability according to SRI cut off value of 80

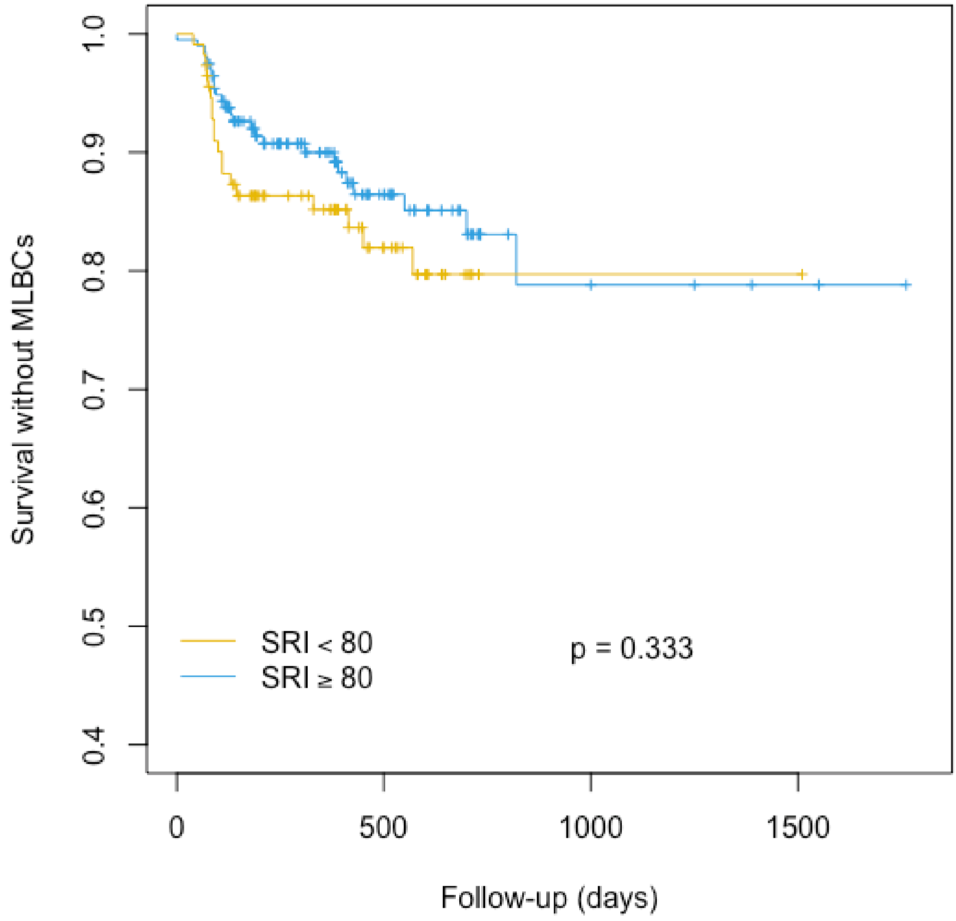

No. at Risk				
SRI < 80	113	31	2	1
SRI > 80	198	54	6	3

Figure legends : SRI : Syntax Revascularisation Index, MLBCs : Major/life threatening bleeding
